# The Effectiveness of EduMind for Mental Health Promotion Among Youths

**DOI:** 10.7759/cureus.61462

**Published:** 2024-05-31

**Authors:** Hazwa Harith, Nik Daliana Nik Farid, Abqariyah Yahya, Nor Liyana Mohd Shuib

**Affiliations:** 1 Department of Social and Preventive Medicine, Faculty of Medicine, Universiti Malaya, Kuala Lumpur, MYS; 2 Department of Information Systems, Faculty of Computer Science and Information Technology, Universiti Malaya, Kuala Lumpur, MYS

**Keywords:** youth mental health, public mental health, digital mental health, public health, mental health

## Abstract

Introduction

The global surge in mental health issues, particularly among the youth, is evident. Despite the proliferation of digital mental health services, their adoption remains limited, hindered by various barriers. To address this issue, an evidence-based, validated digital mental health intervention is necessary. Although much research has explored the effectiveness of such interventions, there was limited evidence supporting those within the youth population. The objective of this research is to assess the effects of an interventional module on depression, anxiety, and stress symptoms among youths.

Methods

The EduMind online mental health intervention contents were developed from a needs assessment and a scoping review of effective psychotherapies, achieving a high content validation index (CVI) of 0.96. The contents were integrated into a web application to assess its effectiveness among the target population which consisted of university students of a local institution.

A quasi-experimental study compared the intervention group (n=264) to a waitlist-control group (n=200), evaluating changes in mental health status with the Depression, Anxiety and Stress Scale 21 (DASS-21) questionnaire using analysis of covariance (ANCOVA) to analyse mean differences.

Results

Participants in the intervention group indicated a significant decrease (*p* < 0.001) in depression, anxiety, and stress. The pre-test and post-test mean scores in the intervention group were 15.81 and 8.97 for depression, 11.46 and 7.02 for anxiety and 14.64 and 6.33 for stress, respectively. In the control group, there was no significant difference between the pre-test and post-test mean scores for depression. However, there was a slight significant reduction for anxiety with pre- and post-test scores of 13.31 and 12.95. Stress mean scores exhibited a slight increase, rising from 13.55 to 14.24.

The final phase revealed significant improvements in mental health status between groups, with significant effect sizes for stress (ƞ_p_^2^ = 0.57, p < 0.001), depression (ƞ_p_^2^ = 0.71, p < 0.001), and anxiety (ƞ_p_^2^ = 0.27, p < 0.001).

Conclusion

The findings contribute to the advancement of technology-assisted health services, facilitating greater uptake among the population. This study utilized a comprehensive module development framework and demonstrated the effectiveness of the expert-guided mental health intervention module. Furthermore, the study suggests potential integration with the National Strategic Plan for Mental Health 2020-2025 and the National Mental Health Policy, proposing the web application as a potential compulsory student screening tool administered by universities. The information gathered by this application could inform future research directions, propelling technological-assisted mental health services to new heights.

## Introduction

According to the National Health and Morbidity Survey 2023: Non-Communicable Diseases and Healthcare Demand (NHMS), approximately one million individuals aged 15 and above were found to be suffering from depression. The survey revealed a twofold increase in the prevalence of depression from 2019 to 2023, notably more pronounced among younger demographics, particularly individuals aged 16 to 29 years old. According to the report, a total of 4.6% (1,000,000) of adults in Malaysia were identified as experiencing depressive symptoms [1]. The prevalence of mental health concerns is rising, especially among individuals aged 12 to 24, leading to various developmental challenges such as poor academic performance, substance misuse, and involvement in violent behaviors [2]. Early detection and intervention are crucial to prevent symptoms from worsening and affecting daily functioning, as well as societal and national productivity [3].

In response to this issue, there has been a significant increase in mental health intervention programs and awareness initiatives targeting early detection and support for youth. Online mental health services have become popular among young people due to increased access to mobile technology and the internet [4].

Recognizing the limitations of traditional mental health services, there has been a shift towards comprehensive youth mental health approaches globally, particularly focusing on individuals aged 12-25 [5-7]. These approaches emphasize developmentally and culturally appropriate design features to address the unique biopsychosocial issues of this age group.

Efforts are underway to adopt co-designed, accessible, and "soft entry" mental health services to minimize barriers and create welcoming environments for youth [8-10], leveraging the accelerated digitalization brought about by the pandemic.

Despite progress, mental health interventions often overlook individuals without formal psychiatric evaluations and diagnosis by mental health professionals [11]. The expansion of internet-based therapies, including games and eLearning, allows users to engage anonymously, easing demand on traditional face-to-face services [12].

Studies examining digital mental health services prioritize assessing information quality, accessibility, and user satisfaction [13]. However, these interventions face limited real-world adoption due to stigma and concerns about their effectiveness [14]. Nonetheless, online platforms have demonstrated potential in overcoming barriers such as stigma in accessing mental health care [15].

The growing availability of digital mental health services emphasizes the need to evaluate their effectiveness. EduMind, a mental health web app with the novelty feature of being developed and validated through expert and end-user assessments that specifically caters to local youths (PhD dissertation: Hazwa Harith. The Development and Implementation of EduMind for Mental Health Education Among Youths; 23 April 2024) now requires effectiveness evaluation as part of its final phase. The EduMind contains a segment on mental health status screening followed by interventional activities surrounding psychoeducation, cognitive behavioral therapy, breathing exercises and mood journaling.

## Materials and methods

Study design and sampling

A quasi-experimental study was conducted on youths aged between 18 - 25 years in a local university, Malaysia. Students with established mental illness and the presence of active psychosis or hallucinations with the use of psychiatric medications were excluded. The participants who fulfilled the inclusion criteria of being within age 18 - 25 years, and has access to mobile internet, were divided into two groups (intervention and waitlist control groups). The study involved a total of 464 participants, divided into two groups: 264 students in the intervention group and 200 students in the waitlist control group who were non-randomly selected. The selection and distribution process were based on the classes that were approached. Entire classes were chosen to participate, and students within each selected class were assigned to either the intervention group or the control group. Utilizing a power of .80 and anticipated effect sizes of d= .36 for depression (as reported by Howells et al., 2016 [16]) and d= .62 for depression/anxiety and stress [17] with 20% attrition rate, the estimated sample size was 154 participants with 77 participants for each group.

Data collection tool

This study has acquired approval from the Universiti Malaya Research Ethics Committee (Ref No.: UM.TNC2/UMREC_1790) and is registered with the Thai Clinical Trials Registry (TCTR) (Reg No.: TCTR20231011002). The TCTR is the primary registry for experiment studies within the Southeast Asia region.

The selection of participants was purposeful in nature, involving the dissemination of information about the study to faculties through official letters and emails addressed to deans and heads of departments. The faculties that responded positively to facilitate the study included the Faculty of Science, Faculty of Education, Faculty of Medicine, Faculty of Engineering, Faculty of Computer Science, and Information Technology, as well as the Faculty of Languages and Linguistics. Lecturers, identified by the heads of departments, were then approached for the enrolment of students of their respective classes. In addition to official enrolments, participants were also recruited through the dissemination of study information via WhatsApp, an online messaging platform. Details were provided to the head of the student representative for the involved faculties. Participants who agreed to participate were given a concise overview of the nature of the study followed by consent taking. Emphasis was placed on aspects related to the privacy and confidentiality of the gathered data, and it was reiterated to the students that participation was entirely voluntary. For those who declined to join, they are encouraged to be aware of their symptoms and seek help with the university’s counselling services should the need arise. According to the sample size calculated, the number of students recruited fulfilled the calculation obtained from the G*Power software which was 154 participants. The students were then allocated into two groups: intervention (264 students) and waitlist-control group (200).

Upon enrolment, the participants were given instructions to access EduMind via the link given and proceed with the succeeding activities. The initial segment of the EduMind web app consisted of a basic sociodemographic questionnaire, followed by a mental health screening survey using the Depression, Anxiety and Stress Scale (DASS-21) [18]. The short form of the DASS-21 survey is composed of three symptom-based subscales which explored the participants’ experience over the past one week. Each subscale comprised seven items, and participants provided responses on a 4-point Likert scale (0=not at all to 3=most of the time). After converting scores to align with the DASS-42, summed scores for each scale range from 0 to 42, where higher scores indicate more severe symptoms. Among Malaysian samples, the DASS-21 has shown reliability with a Cronbach alpha of .87 for depression, .92 for anxiety, and .89 for the stress subscales [19]. Furthermore, the DASS-21 has demonstrated close correlations with Diagnostic and Statistical Manual of Mental Disorders (DSM) diagnoses, including panic disorder, generalized anxiety disorder, social phobia, simple phobia, and major depressive disorder [18,20]. Both groups were given the same screening questions to determine their baseline mental health status using a self-administered technique. To ensure all sections were completed, users could not proceed if any information was missing.

Interventions

Participants were allocated conveniently, with students assigned to groups based on their classes and availability. The intervention group participated in a week-long intervention, while the control group did not have access to the EduMind intervention components. Further elaborations are described in subsequent sections.

Intervention Group

The students conveniently allocated into the interventional group undergo a baseline screening question, that is available in the full EduMind website (full version), consisting of eligibility criteria, sociodemographic background, and a survey to measure their current mental health status. After the completion of the first part, they were then given immediate and direct access to the developed mental health modules which contain elements of psychoeducation, cognitive behavioural therapy, breathing exercises and mood journaling. They were subsequently directed to engage in recommended activities tailored to their individual mental health status and encouraged to interact with the website for a minimum of 10 minutes daily, at least five times a week, aligning with the effectiveness guidelines outlined by Marshall and colleagues [21] to simulate short term mental health interventions. The frequency of web app visits was tracked using self-administered questions at the end of the one-week duration. After one week, the investigator will be present at the same class venue to oversee that the recruited students have completed the follow-up mental health screening questions. Text messages were sent to remind students to answer the follow-up mental health screening survey.

Waitlist Control Group

Students assigned to the waitlist group undergo baseline screening questions available on the limited version of the EduMind website (limited version), covering eligibility criteria, sociodemographic background, and a survey to gauge their current mental health status. Following the completion of the initial segment, they were not given access to the full contents of the EduMind website. However, they were reminded to seek emergency help if they were feeling overwhelmed or suicidal. After one week, the investigator ensured that the recruited students in this group had completed the follow-up mental health screening questions at the same class venue. At this juncture, students in the waitlist group were provided access to the full version of the EduMind website (full version), encompassing recommended activities based on their mental health status scores.

EduMind Contents

The contents of EduMind have been developed using multi-phased procedures consisting of an initial needs assessment (Phase 1), followed by a scoping review (Phase 2) and content validation and incorporation into web app platform (Phase 3). The needs assessment was done to identify refinements and improvements for a digital mental health intervention based on a currently available mental health app. The subsequent scoping review shortlisted suitable content and psychotherapies that were not only evidence-based but also closely adhered to the standard care recommended by mental health experts. The developed contents, drafted from the findings of the prior steps, were then validated among mental and public health experts. The contents, encompassing psychoeducation, cognitive behavioral therapy, breathing exercises and mood journaling, obtained a high content validation index (CVI) of 0.96 and was subsequently embedded into the web application format that was chosen for its versatility and readily available features.

Data analysis

The data collected from participants, encompassing sociodemographic details and scores from the DASS-21 questionnaire, were securely stored on a web development platform. The platform was protected by robust encryption with a password, ensuring exclusive access for the primary investigator. The information available on the platform would be in its raw form and necessitated further classification and processing.

For data analysis in this study, Statistical Package for the Social Sciences (SPSS) version 26 for the Mac Operating System was employed. Developed by the International Business Machines (IBM) Corporation (Armonk, NY, USA), SPSS was selected for several reasons. Its user-friendly interface, the investigator's familiarity with the software, and its widespread use in social sciences for comprehensive statistical analyses contributed to its choice. Additionally, SPSS facilitated the effective management of data obtained from the web platform.

Descriptive analysis was used for sociodemographic data of the participants whereas for analytical tests, paired t-test and analysis of covariance (ANCOVA) were performed to evaluate the effects of the intervention of both the intervention and waitlist control groups. The normality of data was tested using the Kolmogorov-Smirnov test. The p-value was set at <0.05 for statistical significance.

## Results

Sociodemographic characteristics

The characteristics of the participants in the study are shown in Table 1. A total of 464 students agreed and gave their consent to participate and 264 and 200 students were allocated into intervention and control groups respectively. There was a total of 115 (43.6%) and 96 males (48.0%) for each intervention and control group respectively. Subsequently, the intervention group comprised 149 females (56.4%), while the waitlist control group consisted of 104 females (52.0%). The mean age was 20.88 (SD=1.0) for the intervention group whereas for the waitlist control, their mean age was 20.35 (SD=1.2) which corresponds to the targeted age group i.e. youths. The greatest number of students of the intervention group would be Year 1 students (120) and followed by those in Year 4 with 62 students. The remainder were Year 2 students (35), Year 3 students (45) and only two students belonged in Year 5 for the intervention group. Regarding the control group, the distribution of study years was nearly uniform across all categories except for the Year 5 students. Year 1 had 46 students, Year 2 had 58 students, Year 3 had 36 students, Year 4 had 59 students, and Year 5 had only one student. Based on their course background, students allocated into the intervention group were mostly from a science background (86.4%) and those in the waitlist control group mainly comprised those from a non-science background (57.0%).

**Table 1:** Sociodemographic background of participants The data has been represented as N and in percentage (%).

	Intervention Group (N = 264)	Waitlist Control Group (N = 200)
Mean age (SD)	20.88 (1.0)	20.35 (1.2)
Gender		
Males	115 (43.6%)	96 (48.0%)
Females	149 (56.4%)	104 (52.0%)
Year of study		
Year 1	120 (45.5%)	46 (23.0%)
Year 2	35 (13.3%)	58 (29.0%)
Year 3	45 (17.0%)	36 (18.0%)
Year 4	62 (23.5%)	59 (29.5%)
Year 5	2 (0.8%)	1 (0.5%)
Course background		
Science	228 (86.4%)	86 (43.0%)
Non-science	36 (13.6%)	114 (57.0%)

Baseline DASS-21 scores

Table 2 shows the baseline scores for participants in the intervention group where they obtained a moderate score for both depression (mean score = 15.81 ± 6.78) and anxiety (mean score = 11.46 ± 7.85). Stress mean score for this group was 14.64 ± 7.92, which was mild. Based on follow-up data, there was a decrease in scores for students in the intervention group across all three parameters. Specifically, depression scores decreased from 15.81 to 8.97 ± 5.06, anxiety scores showed a reduction from 11.46 to 7.02 ± 5.81, and stress scores declined from 14.64 to 6.33 ± 5.47.

**Table 2:** Baseline mental health status of participants (both intervention and waitlist control groups) * P - value is significant < 0.05 The data has been represented as mean ± SD with p<0.05 set as being significant.

	Intervention Group (N = 264)				Waitlist Control Group (N = 200)			
	Baseline mean score, T_0_	Follow up mean score, T_1_	t	Sig.	Baseline mean score, T_0_	Follow up mean score, T_1_	t	Sig.
Depression (SD)	15.81 (6.78) Moderate	8.97 (5.06) Normal	37.00	<0.001*	12.08 (6.56) Mild	12.08 (6.56) Mild	NA	NA
Anxiety (SD)	11.46 (7.85) Moderate	7.02 (5.81) Normal	12.42	<0.001*	13.31 (7.38) Moderate	12.95 (6.81) Moderate	1.99	<0.001*
Stress (SD)	14.64 (7.92) Mild	6.33 (5.47) Normal	34.60	<0.001*	13.55 (8.29) Normal	14.24 (7.87) Normal	- 1.98	<0.001*

The baseline mean score for the waitlist control group was mild with 12.08 ± 6.56, 13.31 ± 7.38 (moderate) and 13.55 ± 8.29 (normal) for depression, anxiety, and stress respectively (all p < 0.001 except for depression). Within the waitlist control group, the depression score remained consistent across both time points. Meanwhile, there was a slight decrease in anxiety scores, shifting from 13.31 to 12.95 ± 6.81. However, stress mean scores exhibited a slight increase, rising from 13.55 to 14.24 ± 7.87.

Mean difference of DASS-21 scores

Table 3 presents the results of the paired t-test analysis for the mean differences of the mental health measures among participants in the intervention group between the two time points. Users of the EduMind website reported significant reductions in depression, anxiety, and stress, from baseline to the end of the seven-day period. The mean difference for depression, where there was a reduction of scores at the follow-up time point, was reported as 6.84 (SD = 3.00, p < 0.001, 95% CI [6.48 - 7.20]). There were also improvements in anxiety and stress where participants reported a mean difference of 4.45 (SD = 5.82, p < 0.001, 95% CI [3.74 - 5.15]) and 8.31 (SD= 3.90, p < 0.001, 95% CI [7.84 - 8.79]) respectively.

**Table 3:** Mean score difference for intervention group *p<0.05 for within-group differences at postintervention (paired t-test) The data has been represented as mean ± SD with p<0.05 set as being significant.

Intervention group (N= 264)				
	Paired mean score difference (T_0_ vs T_1_)	95% CI	t	Sig.
Depression (SD)	6.84 (3.00)	6.48 – 7.20	37.00	< 0.001*
Anxiety (SD)	4.45 (5.82)	3.74 – 5.15	12.42	< 0.001*
Stress (SD)	8.31 (3.90)	7.84 – 8.79	34.60	< 0.001*

Within the control group (Table 4), participants reported no changes in their depressive symptoms hence the paired t-test analysis showed no mean difference between their baseline reading and at the follow-up time point. However, it was reported that scores for anxiety improved slightly with a mean difference of 0.36 (SD = 2.59, p < 0.001, 95% CI [0.00 - 0.72]). However, there was a reported increment for stress, with a mean difference of -0.69 (SD = 5.14, p < 0.001, 95% CI [-1.41 - 0.03]) with the negative value denoting that the post score was higher than then the scores at baseline.

**Table 4:** Mean score difference for control group *p<0.05 for within-group differences at postintervention (paired t-test) The data has been represented as mean ± SD with p<0.05 set as being significant.

Control group (N=200)				
	Paired mean score difference (T_0_ vs T_1_)	95% CI	t	Sig.
Depression (SD)	0	NA	NA	NA
Anxiety (SD)	0.36 (2.59)	0.00 – 0.72	1.99	< 0.001*
Stress (SD)	- 0.69 (5.14)	- 1.41 – 0.03	- 1.90	< 0.001*

Effectiveness of EduMind

In examining the effects of the EduMind online modules on the symptoms of depression, anxiety and stress of the participants, an intention to treat (ITT) ANCOVA was conducted comparing both groups with covariate adjustment. The covariate of interest, which was the pre-test or baseline scores, was controlled. Levene’s test and normality checks were carried out and the assumptions were met.

The partial Eta Squared value (ηp2) indicates the effect size and should be compared with Cohen’s guidelines (0.2 - small effect, 0.5 - moderate effect, 0.8 - large effect). The results in Table 5 showed that after an analysis with a resultant Bonferroni test, there was a significant difference in mean post-scores between the groups for depression [F (1,461) = 1115.58, p < 0.001, ηp2 = 0.708], anxiety [F (1,461) = 169.86, p < 0.001, ηp2 = 0.269] and stress [F (1,461) = 631.59, p < 0.001, ηp2 = 0.578] while adjusting for baseline scores. Thus, it can be said that the effect size for depression was moderate (0.7), anxiety was small (0.3), and stress was moderate (0.6).

**Table 5:** Between-group comparison of intervention and control group *p<0.05 for between-group differences at postintervention (analysis of covariance (ANCOVA)) ^a^Analyzed using partial eta squared (η_p_^2^) The data has been represented as mean ± SD with p<0.05 set as being significant.

	PRE		POST		Effect within^a^ (time)	Effect between^a^ (time*group)	Sig.	Observed power
	Intervention	Control	Intervention	Control				
	Mean (SD)		Mean (SD)					
Depression	15.81 (6.78)	12.08 (6.56)	8.97 (5.06)	12.08 (6.56)	0.892	0.708	< 0.001*	1.000
Anxiety	11.46 (7.85)	13.31 (7.38)	7.02 (5.81)	12.95 (6.81)	0.623	0.269	< 0.001*	1.000
Stress	14.64 (7.92)	13.55 (8.29)	6.33 (5.47)	14.24 (7.87)	0.693	0.578	< 0.001*	1.000

## Discussion

The final phase of the study focuses on establishing the effectiveness of the online mental health intervention, EduMind. Results from the quasi-experimental study indicate that EduMind significantly improved participants' symptoms, with scores for depression, anxiety, and stress showing significant reductions compared to a waitlist control group. Initially, participants in the intervention group exhibited mild to moderate levels of these mental health issues at baseline. After one week of exposure to the intervention, participants reported improvements, achieving normal mean scores across all mental health problems. Hawthorne effect may be evident here as reported by Sedgwick and Greenwood [22]. Knowledge of the treatment allocation may have contributed to the Hawthorne effect. However, the Hawthorne effect can exist even if a trial is double-blind. To minimize this effect, this study has incorporated the inclusion of control group in which the procedures were consistent with the intervention group. Furthermore, the usage of EduMind, consist of naturalistic environments in terms of observation as the app was encouraged to be used within the comforts of their privacy. Furthermore, to address Hawthorne’s effect, the participants were assured of their confidentiality.

Drawing on existing research, there is a notable gap in the mental health field regarding the exploration of online self-help modules that comprehensively address depression, anxiety, and stress within a single platform. This study aims to fill this gap by providing initial insights into content that could benefit and enhance the mental health of youths. By utilizing the DASS-21 mental health survey, this study demonstrates the overall mental health status of participants, showing significant interrelationships among depression, anxiety, and stress scores. Individuals with higher depression scores tend to exhibit elevated levels of anxiety and stress, and vice versa. This underscores the importance of developing and implementing a self-help program like EduMind that addresses these interconnected issues. Considering this, basic cognitive behavioural therapy (CBT) has been identified as a suitable approach to guide users in managing these interrelated symptoms [23].

The effect sizes for improvements in comparison to the control condition were coherent with the findings made by earlier studies that tested self-help programs as reported by previous studies [17,23,24]. The different range of effect sizes between depression, anxiety and stress may be explained by the nature of the symptoms experienced by the participants. Improvements were seen mostly in depression as compared to similar studies where the greatest effect sizes were seen for anxiety as compared to depression [25]. However, there were previous studies that supported similar notions of the current studies and hence showed greater effect sizes in anxiety rather than depression [26,27]

The larger effect size as seen for depression was probably due to the content of the EduMind online module where most of the interventional strategy utilizes CBT in which it is more applicable to those with depressive symptoms [28]. However, it is crucial to highlight that during the designing and development stage, the incorporation of CBT elements was thoroughly considered to include and address all three mental health problems.

Participants in the intervention group showed improvement within just one week of receiving the intervention. Therefore, with the establishment of a system that ensures or promotes ongoing user participation and engagement, a more substantial improvement is anticipated. This suggests that the EduMind intervention could serve as a valuable alternative to more intensive or costly treatment options, particularly for individuals experiencing comorbid symptoms of depression, anxiety, and stress. Nevertheless, extended follow-up studies are essential to assess the treatment benefits over a more prolonged duration.

The EduMind was designed as a comprehensive mental health program encompassing awareness and intervention. Its primary goal was to enhance the overall mental well-being of users, particularly focusing on the youth demographic due to their various risk factors. The inclusion of a mental health survey, such as the DASS-21, along with symptom descriptions and appropriate activities, contributed significantly to achieving the main objective. Slight reduction in the symptoms experienced within the control group may be due to the awareness that they have obtained from participating in the mental health survey and being exposed to the explanations of their experienced emotions. This provides better clarity in understanding their current mental health status and thus increases their motivation to seek help. The control group was also given access to helplines should there be a need to address any overwhelming and life-threatening situations.

While the enrollment in this study played a significant role in the adoption, it is noteworthy that, with endorsement from the university's higher management, this website could evolve into a primary screening tool for incoming undergraduates. It offers an opportunity for students to delve into and understand their symptoms through the available information on the website. This could serve as a preventive measure and an early form of intervention, aiding in the initial stages of seeking a professional diagnosis from mental health experts.

The widespread adoption of online mental health interventions has the potential to promote greater awareness of mental health among young individuals and enhance their understanding of symptoms. This, in turn, could empower youths with resilience and self-sustaining skills, contributing to their overall well-being. In addressing mental health and well-being, the National Mental Health Policy highlights various roles undertaken by the Ministry of Health Malaysia (MOH). These include establishing a foundation for developing a strategy to guide all stakeholders involved in planning and executing health programs aimed at enhancing the mental health and well-being of the population. Additionally, the policy focuses on enhancing mental health services for populations susceptible to psychosocial challenges, as well as improving psychiatric services for individuals with mental disorders and striving to provide care and support to families, communities, and relevant entities [29].

The policy also underscores a specific program focusing on prevention and treatment for the youth demographic, emphasizing rehabilitation activities. In executing the policies, the government has developed a specific national strategic plan to rectify the growing concerns surrounding mental health issues.

In reference to the recent National Strategic Plan (NSP) for Mental Health 2020 - 2025 [30] it was reported that a comprehensive, national-level policy encompassing strategy and action plans across all sectors and levels of governance is essential. Within the NSP, there is a potential for self-help online mental health intervention such as the EduMind. referencing to the third strategy outlined in the NSP, which is on ensuring the availability and accessibility of comprehensive and quality mental health services through primary, secondary, and tertiary healthcare. The activity detailed was to develop an e-mental screening app that can be highly utilized among tertiary education students and was again reiterated in the sixth strategy which is on promoting mental health and wellbeing in all settings and target groups.

The implementation of digital mental health services such as the EduMind would complement well the existing efforts that are in place. Examples of current services would be the programs within the educational institutions such as counselling services, training of trainers for university staff and student volunteers, and psychological first aid (PFA).

Limitations

This study had a few limitations that probably stemmed from the utilization of quasi experiment study instead of a randomized control trial. Limitations such as non-randomization and blinding can give rise to Hawthorne’s effect. Additionally, a longer follow up can further establish the time relation and effect sizes. Furthermore, this study was limited to a certain population in a local university setting that has given rise to a few biases. To extrapolate the effectiveness of the EduMind, future study with a randomized control design and the inclusion of general youth population are essential to reduce these limitations.

## Conclusions

This quasi-experimental study in determining the effect of EduMind in improving the mental health status among youths was able to establish the effectiveness that was presented with significantly high effect sizes (0.3 for anxiety, 0.6 for stress and 0.7 for depression). It would be the answer to the current gaps by producing an evidence-based, expert-validated digital mental health intervention that is proven to be effective. The findings also demonstrated the importance of having to perform proper development and content validation processes in producing an effective intervention that adheres to the standard level of mental health care. Furthermore, the study suggests potential integration with the Malaysian National Strategic Plan for Mental Health 2020-2025 and the National Mental Health Policy, proposing the web application as a potential compulsory student screening tool administered by universities. The information gathered by this application could inform future research directions, propelling technological-assisted mental health services to new heights.
